# *Streptomyces* cell-free systems for natural product discovery and engineering

**DOI:** 10.1039/d2np00057a

**Published:** 2022-11-07

**Authors:** Simon J. Moore, Hung-En Lai, Jian Li, Paul S. Freemont

**Affiliations:** a School of Biosciences, University of Kent UK; b School of Biological and Behavioural Sciences, Queen Mary University of London UK simon.moore@qmul.ac.uk; c School of Biological Sciences, Victoria University of Wellington New Zealand; d School of Physical Science and Technology, ShanghaiTech University China; e Department of Medicine, Imperial College London UK p.freemont@imperial.ac.uk

## Abstract

*Streptomyces* bacteria are a major microbial source of natural products, which are encoded within so-called biosynthetic gene clusters (BGCs). This highlight discusses the emergence of native *Streptomyce*s cell-free systems as a new tool to accelerate the study of the fundamental chemistry and biology of natural product biosynthesis from these bacteria. Cell-free systems provide a prototyping platform to study plug-and-play reactions in microscale reactions. So far, *Streptomyce*s cell-free systems have been used to rapidly characterise gene expression regulation, access secondary metabolite biosynthetic enzymes, and catalyse cell-free transcription, translation, and biosynthesis of example natural products. With further progress, we anticipate the development of more complex systems to complement existing experimental tools for the discovery and engineering of natural product biosynthesis from *Streptomyces* and related high G + C (%) bacteria.

## Abstract 2

*Streptomyces* cell-free gene expression (CFE) systems for natural product discovery. The CFE system simply requires DNA, a cell-extract, amino acids, and a metabolite solution to study transcription, translation, and biosynthesis in a single reaction.

## Graphical abstract

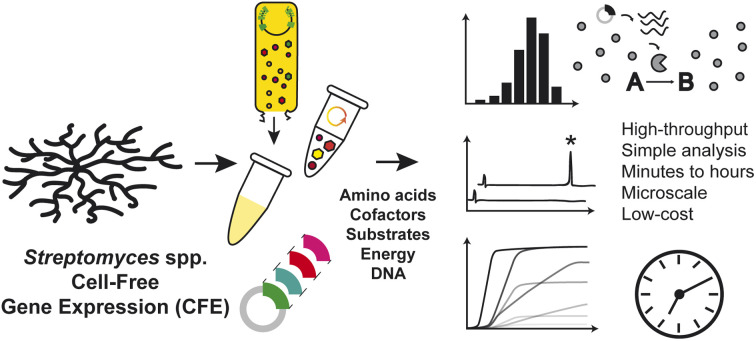

## Introduction

*Streptomyces* is a genus of Gram-positive bacteria, which are a major source of natural products. These environmental bacteria have led to the discovery of hundreds of drugs and commercialised chemicals, including about two-thirds of derived antibiotic scaffolds.^1^ With the rise of genome sequencing,^2^ there is renewed vigour for studying the fundamental chemistry and biology of *Streptomyces* and the natural products that they produce.^3,4,5^ However, it is challenging to study and engineer *Streptomyces* to access new natural products. This review will discuss the emergence of specialised *Streptomyces* cell-free lysate systems and how these platforms help complement existing experimental and computational approaches in natural product discovery. The motivation for developing *Streptomyces* cell-free lysate systems is the potential to study gene expression regulation, enzymes and biosynthetic pathways encoded within so-called “silent” or “cryptic” biosynthetic gene clusters (BGCs) native to *Streptomyces* genomes.^6^ We will first summarise existing approaches within the natural products field, before discussing current and potential future applications of *Streptomyces* cell-free lysate systems.

## Natural product discovery from *Streptomyces*

*Streptomyces* are found in terrestrial and aquatic environments, whose genomes have a high guanine and cytosine (G + C) content ranging between 67 to 72%.^5^ Historically, laboratory fermentation of environmental microbes, including *Streptomyces*, inspired a golden era of antibiotic discovery during the 1940–1960s.^6^ Shortly after, rediscovery of antibiotics and the rise of antimicrobial resistance initially suggested that this discovery pipeline was exhausted.^7^ However, in the early 2000s, genome sequencing of the model *Streptomyces coelicolor* A3(2) strain revealed clusters of DNA encoding proteins with similarity to known biosynthetic enzymes.^8^ These co-localised regions were recognised as BGCs linked to natural product biosynthesis. Of the 20 BGCs identified, five produced known natural products.^8^ The remaining BGCs are silent, in terms of gene expression, under standard laboratory conditions.^6,8^ With the rapid rise of next-generation DNA sequencing, there is now a rich availability of genomic and metagenomic sequences from diverse habitats.^3,4^ Specifically, *Streptomyces* genomes (∼6 to 11 Mb) can encode up to 100 BGCs that vary greatly in size from approximately 5 to 100 kb of DNA. Together with multi-omics data,^4,9,10^ new natural product classes,^11^ and improved computational BGC prediction and annotation tools,^3,12^ these combined advances have enabled the prediction of natural product scaffolds based on input DNA sequence – an approach called genome mining.^13^ This is especially true for well-defined natural product classes such as polyketides, non-ribosomal and ribosomal peptides. Although, there is always scope to discover novel natural products from understudied biosynthetic pathways not accurately predicted by current algorithms.^14^ Because of the extensive diversity of BGCs available to study, the prioritisation of novel and/or potentially bioactive BGCs is also an important focus.^4^ However, there is a disparity between the number of predicted BGCs and experimentally characterised natural products. From bacteria alone, the number of chemical classes documented in the Natural Product Atlas^10^ (currently ∼12 500) is approximately 3% of the total predicted potential classes.^4^ Therefore, there is a continuous need to develop higher throughput and better experimental tools to drive new discoveries in natural products.

## Current microbial cell approaches to study natural products encoded by BGCs

Most BGCs are either transcriptionally or translationally silent in terms of gene expression when studied in the laboratory environment.^6,8,15^ Therefore, there are two general approaches to study natural products encoded by BGCs – native or heterologous expression. The traditional approach is the “one strain many compounds” (OSMAC) method to study BGCs that are naturally active in native hosts under laboratory conditions. This involves culturing environmental bacterial or fungal isolates under a wide range of conditions to induce BGC gene expression and natural product biosynthesis. This is because conditions that imitate environmental factors can serendipitously activate “silent” BGCs through unknown gene expression regulators.^16^ Other OSMAC approaches included small molecule elicitor library screening^17^ and co-culturing,^18^ which can simulate complex microbiome interactions in native microenvironments to trigger BGC gene expression and natural product biosynthesis.^18^ However, the OSMAC approach is best applied to novel strains, since key limitations include rediscoveries due to screening bias towards common targets (*i.e.*, antimicrobials), while many BGCs will remain switched-off in terms of gene expression.^19,20^ Most often, the environmental triggers that activate BGC gene expression are absent under laboratory conditions, requiring extensive screening experiments to mimic these triggers fortuitously.

Alternatively, heterologous expression represents a major approach on activating silent BGCs.^19^ There are multiple genetic engineering strategies used to isolate or engineer target BGCs from source (meta)genomes – for more detail on this subject, we recommend the following review.^20^ Although successful in expressing a wide range of natural products and complex BGCs that are otherwise not produced in native hosts, heterologous expression remains a painstaking, trial-and-error process, as it is difficult to predict the optimum expression host and culture condition for each specific BGC.^21^ In addition, many BGCs expressed in heterologous hosts, remain silent in terms of gene expression.^22^ This means further genetic manipulation and metabolic engineering is often required to activate silent BGCs in heterologous hosts.

**Fig. 1:**
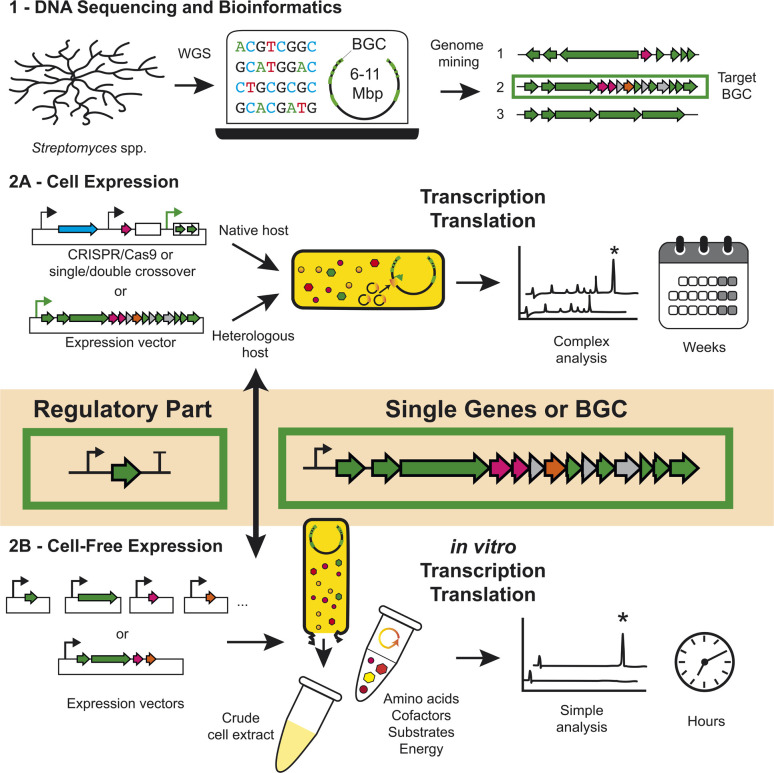
A comparison of genetic engineering approaches to study and engineer BGC from *Streptomyces* (1) First, genomic DNA isolation, DNA sequencing and bioinformatics analysis is required to annotate predicted BGCs. (2A) Cell-based gene expression is the dominant approach used to either directly engineer a BGC in the native host, through recombination cloning methods or through heterologous expression of the BGC in a suitable host strain. (2B) Alternatively, this highlight introduces the concept of *Streptomyces* CFE systems for the study and engineering of BGCs and regulatory components from *Streptomyces* and related bacteria. Potential factors for considering either approach is the complexity of downstream analysis and experimental timeline.

Some of the problems discussed with studying BGC expression – either native or heterologous – are often associated with the inherent challenges of working with complex biological systems. This includes unknown genetic regulation, poor genetic tools, variable product titres and complex metabolite profiles. Herein, this highlight aims to discuss an emerging cell-free synthetic biology tool that bypasses some of the limitations discussed previously (Fig. 1). We will first discuss what a cell-free system is, before focusing on the development of specialised *Streptomyces* cell-free gene expression (CFE) systems as a tool to study the biology and chemistry of natural product biosynthesis from *Streptomyces*, and related high G + C (%) bacteria.

## Cell-free synthetic biology

Cell-free systems are an emerging platform technology within synthetic biology – a sub-field referred to as cell-free synthetic biology. Rapid advances within the past two decades have demonstrated potential for cell-free systems to compete or replace traditional cell-based approaches.^23,24^ Specifically, cell-free systems allow the study of enzyme biosynthesis within a test tube, an approach that draws parallels to total synthesis from organic chemistry. In comparison, cell-free systems start from known substrates and proceed to completion through exhaustion of substrates or the accumulation of inhibitory products in batch reactions. Since the reactions are biological, reactions do not require elevated temperatures, organic solvents, or heavy metal catalysts, except for some metalloenzymes. To extend reaction time, scaled-up continuous or fed-batch methods can increase the duration, efficiency and yields of reactions.^24^ At this point, we highlight that there are two distinct cell-free approaches. First, enzyme pathways can be assembled in a single “one-pot” biosynthetic reaction, using purified enzymes or crude cell extracts, combined with cofactors and substrates. For this, the key advantage of cell-free “one-pot” systems provide is the ability to make complex natural products, or toxic intermediates, that are challenging to make within a cell, or to probe specific chemical mechanism questions. Although not covered in detail within this highlight, we refer the reader to some recent literature reviews.^25,26^ We will instead focus on discussing the emerging crude extract-based *Streptomyces* CFE systems – also called cell-free transcription-translation or cell-free protein synthesis – for studying and engineering natural product biosynthesis. We will discuss specific advantages and the potential of these systems compared to traditional cell-based expression.

CFE systems require a cell extract, DNA, energy, and amino acids to catalyse coupled messenger RNA (mRNA) and protein synthesis in a one-pot reaction.^27,28^ Early CFE studies used cell extract and a synthetic energy regeneration system (*e.g.*, creatine phosphate), along with purified mRNA or a T7 bacteriophage RNA polymerase system, to make recombinant proteins. Importantly, CFE systems are devoid of cell wall, cell membrane, and genomic DNA, which provides specific advantages in comparison to cell-based expression, such as resistance to toxic intermediates and products.^24^ In addition, the crude cell extract processing steps deplete certain metabolites and mRNA carried over from cell growth. This creates a quasi-chemical reactor system, where individual chemical or physical parameters can be fine-tuned to make mRNA, protein, or chemicals as required. Cell extracts are also more complex than originally thought. This is because a bacterial cell extract contains not only the essential components to perform coupled transcription–translation, but also for other metabolic pathways. For protein synthesis alone, over 100 distinct proteins/RNA components are required.^24,27^ This includes RNA polymerase (α_2_ββ′ω), sigma factors, translation factors, 20 aminoacyl-transfer RNA (tRNA) synthetases, 30S/50S ribosomal subunits and tRNA (*i.e.*, 83 in *E. coli*). Although poorly characterised and variable between different cell extract types, a few hundred proteins essential to cell growth are also present. This includes primary metabolic enzymes involved in energy and amino acid biogenesis, as well as vital supporting processes such as RNA degradation, transcription factors and protein folding chaperones. To activate the cell-free systems to make RNA and protein, the reactions require both a primary and secondary energy source.^27^ The primary energy source is nucleotide triphosphates, which drive initial mRNA and protein synthesis. The secondary energy source is required to regenerate the primary energy source from a high-energy substrate, such as glucose, l-glutamate or certain glycolysis or Krebs cycle intermediates.^27–29^ This is important since many of the native primary metabolic pathways are active in cell extracts. For example, catabolism drives adenosine triphosphate (ATP) regeneration from a secondary energy source (*e.g.*, glucose, 3-phosphoglycerate) to leverage maximal protein synthesis.^30^ Importantly, many metabolic enzymes present in the cell extract have phosphorylase activity, which contributes to overall energy loss in the cell-free systems. In a recent study, approximately 98% of total energy (adenosine triphosphate) was shown to be lost to waste.^29^ This is a key limitation within CFE systems; therefore, energy regeneration is a major focal point for CFE optimisation. Finally, primary metabolites, and the free energy generated by catabolic metabolism, provide the basic building blocks of secondary metabolism, which we refer to as natural products. Therefore, there is potentially an untapped capacity of cell-free systems to fuel secondary metabolite biosynthesis. For example, background metabolic pathways could also provide electrons for redox cofactors (*e.g.*, nicotinamide adenine nucleotide, flavins), amino acids for peptide biosynthesis, as well as acetyl-CoA, malonyl-CoA, and related analogs for specific polyketide biosynthetic pathways. We will now review the recent advances in the development of specialised *Streptomyces* CFE systems, as a new tool to study the chemistry and biology of *Streptomyces*.

## *Streptomyces* cell-free gene expression (CFE) systems

Recently, there has been a rising interest in *E. coli* CFE systems to study enzymes and biosynthetic pathways from secondary metabolism.^31^ This is because *E. coli* is the most well-characterized CFE system and capable of making high recombinant protein yields up to 4 mg mL^−1^ in batch reactions.^27,30^ Alternatively, a range of *Streptomyces* CFE systems have emerged, compatible with multiple strains,^32^ for high-yield protein production.^32–37^ Currently, *Streptomyces venezuelae* and *Streptomyces lividans* CFE systems have been shown to reach up to 0.45 mg mL^−1^^35,37^ and 0.52 mg mL^−1^,^33,38^ respectively. These protein yields are close to commercial *E. coli* cell-free systems, which were developed over several decades of research and include many genetic changes to increase recombinant protein yields. While still in development, we suggest the *Streptomyces* CFE systems present several opportunities over the better characterised *E. coli* CFE systems. This includes the provision of a native protein folding environment, high G + C (%) tRNA pool and background metabolism, which may contain enzymes, cofactors or metabolites required for specific natural product biosynthetic pathways. For example, polyketide and non-ribosomal peptide biosynthesis is catalysed by large modular enzymes, which contain an acyl or peptidyl carrier protein (ACP/PCP) domain, respectively. The ACP/PCP domain is post-translationally modified by specialised phosphopantetheinyl transferase (PPTase) enzymes, found only in microbes that make these natural products. While this causes an issue for heterologous expression systems, such as *E. coli*, the *Streptomyces* cell-free extracts may contain PPTase enzymes to activate ACP/PCP domains, although further work is required. On this point, there are also wider opportunities to work with other *Streptomyces* and related actinobacteria strains, especially those with poor genetic tractability. This is because several other *Streptomyces* strains have also been used to establish CFE systems using the same preparation procedure.^32^ These recent studies have provided user-friendly protocols,^32–39^ which will likely lead to the development of more productive *Streptomyces* CFE systems in the future for natural product research. We will now discuss the current advances for these specialised CFE systems, before summarising the next steps for their continued and wider use.

## Regulatory factors controlling BGC gene expression

**Fig. 2:**
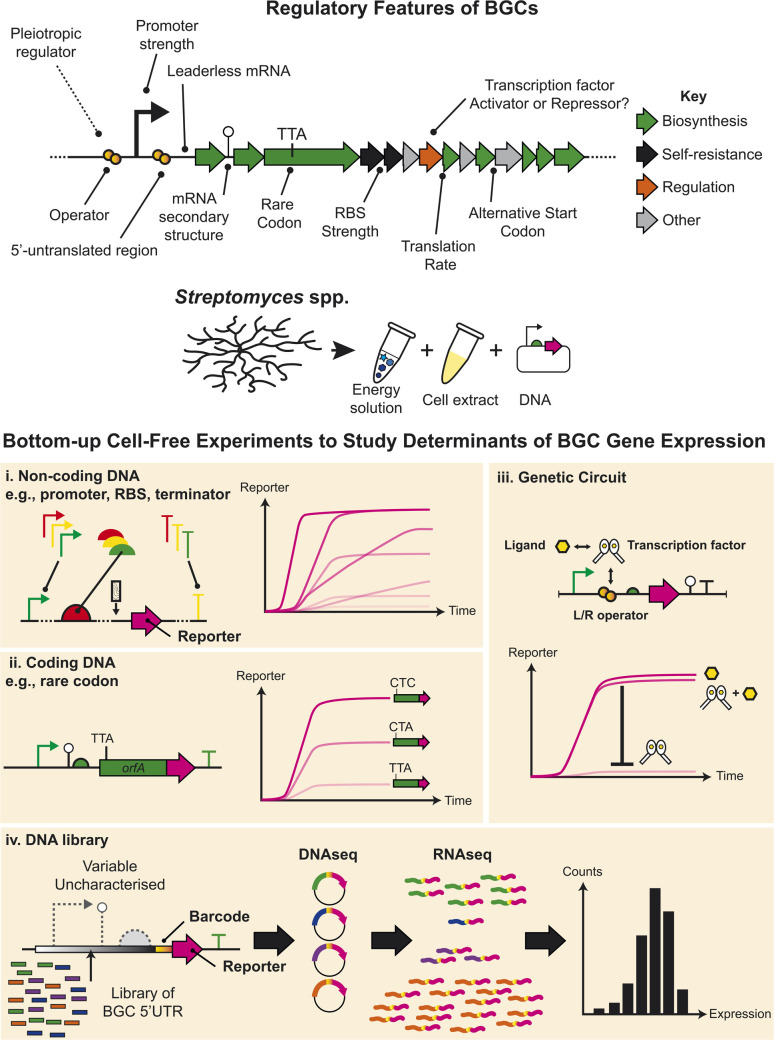
A hypothetical BGC and the regulatory factors that can control gene expression. *Streptomyces* CFE systems offer an opportunity to study individual native regulatory components native from the bottom-up. Potential cell-free experiments include the study of regulatory factors in (i) non-coding and (ii) coding regions of DNA,^33,37^ and (iii) transcription factors.^34^ (iv) CFE also offers the potential to study regulatory components at high-throughput scale, such as the 5′-untranslated region (5′-UTR) of BGCs, which was studied using next-generated sequencing.^43^

It is important to study the fundamental mechanisms of gene expression regulation in order to discover new natural products encoded by BGCs. Focusing on *Streptomyces*, the genetic determinants that control BGC gene expression occur at the level of transcription^40^ and post-transcription regulation, which are highly complex in comparison to better characterised models (Fig. 2). This includes both non-coding and coding DNA sequences that regulate these levels of gene expression. As an example of this complexity, one recent study in *Streptomyces coelicolor* A3(2) showed that RNA and protein levels are not necessarily correlated for secondary metabolism genes, due to efficient translation of even low levels of mRNA.^41^ Other regulatory control points also include alternative start codons,^37^ pleiotropic regulatory factors,^42^ and other regulatory elements, which are summarised in Fig. 2. In addition, unlike better characterised models, the fundamental biological numbers that control protein synthesis, are largely uncharacterised in *Streptomyces*, bacteria. We envision that CFE provides an emerging opportunity to help address some of these questions, not only in *Streptomyces*, but potentially other related actinobacteria and microbial sources of natural products. This is possible because CFE systems are emerging in a range of microbial strains and enable to study of native gene expression regulatory elements from these hosts. A key point of interest in natural product discovery has been refactoring BGCs with optimised regions of non-coding DNA. One example is using strong constitutive promoters to drive gene expression, such as the recent study showing the activation of a “cryptic” streptophenazine BGC in a marine actinobacteria.^44^ This is important within the context of synthetic biology since a repository of characterised DNA parts is a prerequisite for engineering cells. Here, CFE can rapidly characterise regulatory DNA parts in hours, which with the assistance of computational modelling, has been shown to provide strong correlation to cell-based data.^29^ In contrast to slow-growing or genetically intractable strains of *Streptomyces*, the equivalent experimental timeframe is weeks to months (Fig. 2). As discussed, the *Streptomyces* cell extracts contain all the core proteins to catalyse transcription and translation. Another complexity here is that each strain of *Streptomyces* contains between 50–100 Sigma factors, compared to *E. coli* that has seven. The main *E. coli* Sigma factor is σ^70^, which is active during exponential growth to regulate a range of essential genes. The equivalent homolog in *Streptomyces* is HrdB, which is also uniquely post-translationally modified to activate its function.^40^ Interestingly, the core HrdB recognition sequence is homologous to *E. coli* σ^70^ promoters. Originally, a strong *Streptomyces* HrdB-dependent promoter, called kasOp*, was developed with 20-fold more active than the widely used ermEp* promoter.^40^ From this DNA sequence, a synthetic promoter (SP1-SP44) library was created and tested in *S. venezuelae* cells, along with a range of synthetic ribosome-binding site (RBS) parts. This created a diverse combinatorial library with activity up to 1000-fold dynamic range.^45^ The strongest promoter, SP44, was two-fold more active than kasOp*. Two recent CFE studies characterised a selection of this promoter and RBS library in *S. venezuelae* ATCC 10712^37^ and *S. lividans*.^33^ Findings in these two distinct studies were remarkably similar: the SP44 promoter was the strongest in both studies, proving even stronger than the popular T7 RNA polymerase bacteriophage system used widely in biotechnology.^33^ Furthermore, we can also modularly control translation of proteins using RBSs with different strengths.^33,37^ Importantly, CFE plasmids can be constructed with a series of promoters and RBSs to control gene expression levels. Therefore, co-expression of enzymes from a natural product gene cluster can be fine-tuned using different regulatory parts to balance the metabolic flux for pathway optimization. Another advantage of CFE systems is compatibility with high-throughput experiments, such as next-generation sequencing (NGS). Park *et al.* showed a CFE NGS approach, to decode a library of non-coding 5′UTR regulatory sequences associated with predicted BGCs.^43^ A library pool was rapidly characterised both in CFE and *in vivo*, which showed strong positive correlation for these regulatory features. This experiment revealed a snapshot of potential BGCs that are likely transcriptionally active in a specific cell extract. A potential extension of this approach is a diverse selection of *Streptomyces* cell extracts that might provide a screening platform to identify a suitable host cell for heterologous expression.

In addition, CFE also provides the ability to study coding regulatory features (Fig. 2). For example, there is an abundance of alternative start codons in BGCs, which although context dependent (*i.e.*, different between genes/non-coding sequences), is likely to play a key control point for translational efficiency along with the 5′-untranslated region (5′UTR) and initial coding sequence of BGC genes. The *S. venezuelae* CFE system showed that GTG is a weak alternative start codon, while ATG and TTG were equivalent in activity.^37^ Last, there is limited literature concerning how protein synthesis is controlled or can be optimised for *Streptomyces*. For this specific question, *Streptomyces* CFE can be used to study individual rate limiting factors during protein synthesis.^38^

## Natural product biosynthetic enzymes and pathways

While there has been a rising interest in using *E. coli* CFE systems for studying natural product biosynthetic pathways,^46^ the *Streptomyces* CFE systems potentially provide new advantages. Recent examples of *Streptomyces* CFE applied to natural product biosynthesis include the synthesis of a range of oxytetracycline biosynthetic enzymes,^34,37^ single module non-ribosomal peptide synthetase (NRPS) domains^37,38^ and combined transcription, translation and enzyme biosynthesis of some model enzyme pathways from haem and melanin biosynthesis.^37^ A potential advantage for *Streptomyces* CFE is the production of correctly folded and soluble biosynthetic enzymes for biochemical characterisation and structural studies. For example, a large proportion of predicted BGCs encode multidomain type I polyketide synthases (PKS) and NRPS enzymes. These so-called megasynthases can reach over a megadalton in size. Here, open questions remain on how bacterial ribosomes can even make such colossal proteins. Therefore, CFE potentially provides a tool to help fold and solubilise either single or multidomain NRPS/PKS enzymes. Currently, structural biology studies of NRPS/PKS enzymes rely on truncated modules using conventional *E. coli* recombinant protein production, while computational modelling also helps predict how large NRPS/PKS assembly lines are likely folded. So far, *Streptomyces* CFE systems have successfully produced significant yields (microgram level) of NRPS enzymes from valinomycin and tambromycin pathways,^46^ and an uncharacterised single domain NRPS (NH08_RS0107360) from *Streptomyces rimosus*.^37^ Interestingly, *Streptomyces* CFE systems can improve the solubility of proteins originated from *Streptomyces* when compared to *E. coli*-based CFE.^34,39,47^ This could be attributed to the presence of some yet unknown accessory proteins (*e.g.*, molecular chaperones) or metabolite factors in *Streptomyces* cell extracts. Another reason could be that a strain-specific physicochemical environment (*e.g.*, metabolites) that mimics the native *Streptomyces* cytoplasm may play a role in protein folding and solubility.

**Fig. 3:**
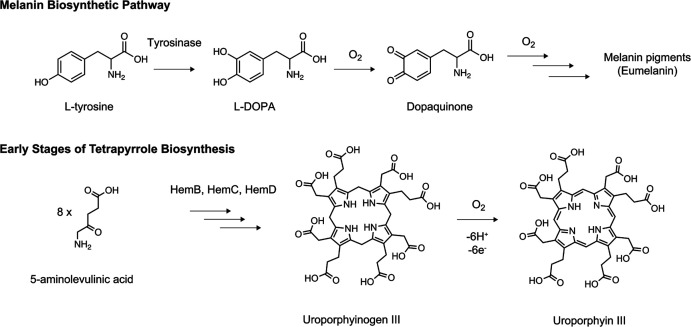
Example biosynthetic pathways produced using *Streptomyces* CFE including melanin and haem biosynthetic intermediates.^37^ Reproduced with permission from ref. 37. Copyright 2021 American Chemical Society.

In terms of *Streptomyces* biosynthetic pathways, three pigmented proof-of-concept examples of combined cell-free transcription, translation, and biosynthesis within a single reaction have been shown.^37^ A simple example is the activity of the β-glucuronidase enzyme from *E. coli* codon-adapted for expression in *S. venezuelae* CFE system. Moving a step further, a two-gene *S. venezuelae* operon encoding tyrosinase and its metallochaperone “caddy” protein were activated, after the addition of copper, post-translation. Here, copper-dependent tyrosinase activity leads to the accumulation of 5,6-dihydroxyphenyalanine that tautomerises to the reactive dopaquinone, which then spontaneously reacts to form a range of melanin pigments (Fig. 3). Last, an operon encoding the early stages of haem biosynthesis to uroporphyrinogen III, the precursor of all tetrapyrroles, was active in the *S. venezuelae* CFE system (Fig. 3). Interestingly, these reactions could be improved by scale-up in a semi-continuous dialysis reaction to afford the uroporphyrin III product in the metabolite feed.^37^ However, both haem and melanin are actively produced during *S. venezuelae* cell growth; haem is an essential molecule for cell growth, while melanin synthesis occurs under specific growth conditions (*i.e.*, mannitol soya flour). Therefore, gene expression regulation is not a limitation for these biosynthetic pathways. In contrast, the study of genes from “silent” BGCs may require further promoter/RBS refactoring and codon optimisation. With further development, native *Streptomyces* CFE systems could provide a rapid prototyping environment to optimise gene expression and biosynthetic pathways.^24^

## Future opportunities

With further developments, both *Streptomyces* and *E. coli* CFE systems provide an alternative and new tool to study uncharacterised BGCs encoding enzymes and biosynthetic pathways from natural product biosynthesis. Specifically, CFE systems offer potential to couple primary metabolism – through cell-lysate energy and cofactor regeneration – with natural product biosynthetic pathways. It is also easier to control metabolites and feed precursors or analogs (*e.g.*, non-canonical amino acids) in a CFE system, whereas cell-based approaches are both time- and resource-consuming to engineer, as well as challenging to achieve consistent production titres. Furthermore, it is desirable to make natural products in a cleaner metabolite background since the chromatography fractions of organic extracts from cultivated *Streptomyces* and other environmental microbes are complex and often challenging to deconvolute. Perhaps the most striking natural product class with immediate potential for CFE systems is the ribosomally-synthesised and post-translationally modified peptides (RiPPs). RiPPs are suitable because the BGCs are relatively small yet encode remarkable chemical and structural diversity through a plethora of post-translational modifications. In addition, RiPP precursor peptides are notoriously challenging to solubilise, wherein CFE systems may provide an advantage. So far, *E. coli* CFE has been used to make diverse RiPP libraries, including combinatorial peptide libraries. This includes improved antibiotic variants of nisin^48^ and millions of sequence-diverse lasso peptides.^49^ There is an abundance of RiPPs in *Streptomyces* and related genomes, and therefore, a well-suited target for the *Streptomyces* CFE systems described. Here, other technologies, such as automation, microfluidics, and machine-learning models, can complement CFE approaches.

**Fig. 4:**
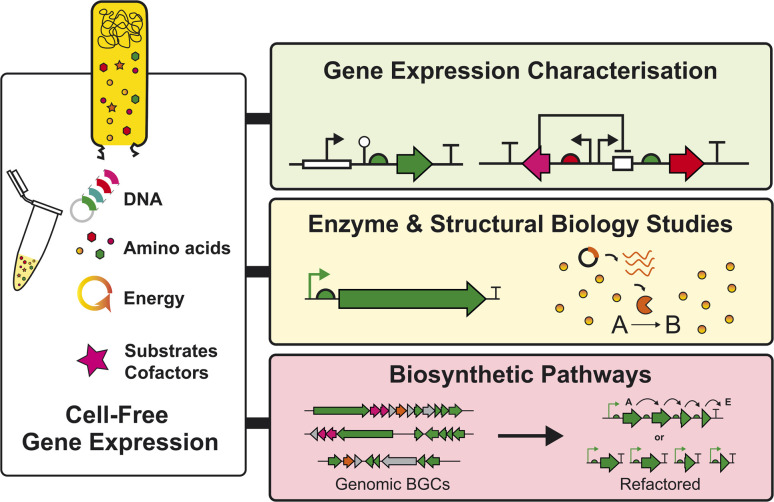
Summary of opportunities for *Streptomyces* CFE systems in the context of natural product biosynthesis and regulation. The CFE reactions require a cell extract, amino acids, energy, DNA, and some additives. This is a plug-and-play chemical reaction, which can be tailored for the desired experiment, covering the levels of mRNA, protein, or small molecule biosynthesis.

A key priority for *Streptomyces* CFE systems is the need to increase protein yields, potentially to levels equivalent or exceeding *E. coli* CFE systems. This will enable further progress and the ability to study more challenging targets (*i.e.*, NRPS/PKS enzymes). For this to occur, it is essential to characterise the precise proteins and metabolites present in the cell extracts, using different analytical methods. While this will differ substantially between extracts obtained from different growth stages, conditions, and strains, it can be used to inform our overall understanding of how we can improve key bottlenecks in CFE systems. These bottlenecks may include regeneration of energy or the supply of biosynthetic precursors, which will be important for optimising natural product titres in CFE systems. Moreover, if specific limiting factors can be identified in the gene expression machinery,^38^ then overall productivity may also be improved through both upstream and downstream engineering strategies. For instance, strain engineering (*e.g.*, modifying nuclease and protease genes), bio-process engineering (*e.g.*, high cell density cultivation in large-scale bioreactors), and reaction engineering (*e.g.*, scale-up, semi-continuous, and continuous reaction modes) are achievable with CFE systems.^24^ Another important focus for *Streptomyces* CFE will include implementing the systems to study the genetic basis of “silent” or “cryptic” BGCs^6^ and the scalability of CFE systems, to make specific proteins for enzymology or structural biology studies^37,39^ and natural products^37^ to complement existing tools in studying natural product biosynthesis (Fig. 4).

## Conclusions

In summary, the key strengths of *Streptomyces* CFE systems are the increased throughput and dexterity to explore a range of biosynthetic or gene expression regulatory questions specific to natural product biosynthetic pathways associated with these specialist environmental bacteria (Fig. 4). Understanding gene expression in these microbes is a fundamental question, and one that if addressed, is likely to lead to new developments in natural product discovery. So far, *Streptomyces* CFE systems have specifically allowed the study of native DNA regulatory features associated with BGCs. This concept can be expanded to other *Streptomyces*, related actinobacteria,^32^ or wider microbial producers of natural products (*e.g.*, *Myxococcus*, *Burkholderia*). Alternatively, the expression of single genes or pathways from metagenomic targets is another attractive target for CFE. This is beneficial, since CFE does not require any genetic manipulation and native CFE systems may provide additional metabolites and cofactors that are not provided in hosts such as *E. coli*. In addition, CFE systems provide a reduced metabolite background,^37^ which with further progress, could be advantageous for the study and isolation of more complex natural product biosynthetic pathways. Currently, it is unrealistic to use CFE systems to express large (>20–100 kb) uncharacterised BGCs, particularly those that encode large modular megasynthases that are still difficult to activate in cell-based heterologous expression. Here, there is a limit to energy exhaustion and substrate depletion in the CFE systems during the entire reaction, due to the increased need for nucleotides, energy, and amino acids for larger BGCs/NRPS/PKS pathway targets. Possible solutions might include developing fed-batch CFE reactions,^37^ which have proved remarkably efficient and scalable for large-scale recombinant protein production.^24^ Finally, it is currently challenging to use CFE to study completely novel BGCs that may encode uncharacterised enzymes or require bespoke cofactors or substrates, although this is also a challenge for cell-based expression. One example is the diverse radical S′-adenosyl-l-methionine (SAM) enzyme family that are challenging to study and require anaerobic conditions. Instead, CFE is potentially better suited for the production and study of single NRPS/PKS enzymes, RiPPs and some well-defined biosynthetic pathways. Also, promiscuous enzymes or biosynthetic pathways that allow the combinatorial feeding of substrate analogs to make new-to-nature natural products, is also an advantage for CFE, where rapid screening is possible.^43,49^ This is advantageous to CFE systems compared to cell-based systems, as CFE systems lack a cell membrane, which may hinder substrate transport. In addition, CFE systems are resistant to substrate/product toxicity issues and can be studied in microscale conditions, which is advantageous for increased throughput and reduced cost. In conclusion, *Streptomyces* CFE systems are an emerging development within synthetic biology that complements and advances existing cell-based expression approaches for natural product discovery.

## Author contributions

All authors contributed equally to the preparation and writing of the review.

## Conflicts of interest

There are no conflicts to declare.

## Supplementary Material
